# Emulsification and pH Control for Sustainable Thermochemical Fluids Reactivity

**DOI:** 10.3390/molecules29225252

**Published:** 2024-11-06

**Authors:** Ali A. Al-Taq, Murtada Saleh Aljawad, Olalekan Saheed Alade, Hassan M. Ajwad, Sidqi A. Abu-Khamsin, Shirish Patil, Mohamed Mahmoud

**Affiliations:** 1 Department of Petroleum Engineering, King Fahd University of Petroleum & Minerals, Dhahran 31261, Saudi Arabia; alialtaq@hotmail.com (A.A.A.-T.); skhamsin@kfupm.edu.sa (S.A.A.-K.); patil@kfupm.edu.sa (S.P.); mmahmoud@kfupm.edu.sa (M.M.); 2 Center for Integrative Petroleum Research, King Fahd University of Petroleum & Minerals, Dhahran 31261, Saudi Arabia; 3 Research and Development Center, Saudi Aramco, Dhahran 31311, Saudi Arabia; haa203@gmail.com

**Keywords:** thermochemicals, emulsification, controlled reactivity, sustainability

## Abstract

Managing chemical reactivity is crucial for sustainable chemistry and industry, fostering efficiency, reducing chemical waste, saving energy, and protecting the environment. Emulsification is used for different purposes, among them controlling the reactivity of highly reactive chemicals. Thermochemical fluids (TCFs), such as NH_4_Cl and NaNO_2_ salts, have been utilized in various applications, including the oil and gas industry. However, the excessive reactivity of TCFs limits their applications and consequently negatively impacts the potential success rates. In this study, an emulsification technique was employed to control the high reactivity of TCFs explored at 50% and 70% in diesel, using three distinct emulsifier systems at concentrations of 1%, 3%, and 5% to form water-in-oil emulsions. The reactivity of 4M neat TCFs and emulsified solutions was examined in an autoclave reactor as a function of triggering temperatures of 65–95 °C, volume fraction, and emulsifier type and concentration. Additionally, this study explores an alternative method for controlling TCF reactivity through pH adjustment. It investigates the impact of TCFs at pH values ranging from 6 to 10 and the initial pressure on the resulting pressure, temperature, and time needed to initiate the TCF’s reaction. The results revealed that both emulsification and pH adjustment have the potential to promote sustainability by controlling the reactivity of TCF reactions. The findings from this study can be utilized to optimize various downhole applications of TCFs, enhancing the efficiency of TCF reactions and success rates. This paper presents in detail the results obtained, and discusses the potential contributions of the examined TCFs’ reactivity control techniques to sustainability.

## 1. Introduction

Thermochemical fluids (TCFs) are widely used across various industrial sectors, including the petroleum industry. Because of their relatively low cost, significant heat generation, and environmentally friendly byproducts (N_2_, NaCl, and H_2_O), ammonium chloride (NH_4_Cl) and sodium nitrite (NaNO_2_) are among the most commonly utilized TCFs in the oil industry. They are highly reactive chemicals, and their exothermic chemical reaction is described by the following equation [1,2,3]:

NaNO_2_ + NH_4_Cl → NaCl + 2H_2_O + N_2_↑; (ΔHRx = −79.95 kcal mol^−1^) (1)

This irreversible reaction has an equilibrium constant of *K_eq_* = 3.9 × 10^71^ Pa × mol × m^−3^ at 25 °C [1]. The high reactivity of this TCF system limits its applications, indicating the need to explore different methods to control its reactivity. Al-Taq et al. [4] demonstrated that the reaction rate of a TCF system is significantly influenced by the pH values of the system and the triggering temperatures. These findings suggest that the reactivity of the TCFs can be tailored to meet various application requirements. Generally, the reaction rate of a chemical reaction is influenced by reactant concentration, temperature, catalysts, and inhibitors. For a reaction to occur, the activation energy must be overcome either by catalysts or by providing the necessary energy.

Physical and chemical methods can be used to control a chemical reaction. Thermochemical reactions are classified as fused reactions when they are delayed [2]. Nguyen et al. [2,3] discussed several techniques to delay heat release from typical TCFs (NH_4_Cl/NaNO_2_), including chemical fuses and physical methods. The chemical fuses include series reactions, autocatalytic reactions, and retardants. The physical techniques include physical separation, encapsulation, and emulsification. Unlike in oil production operations, where emulsion is usually presented as a problem, emulsion may be presented as a solution in several fields, including food, medicine, and detergent industries. Accordingly, emulsification also provides an effective method to control the reactivity of highly reactive chemicals in the oil industry. Emulsification of individual components of the NH_4_Cl/NaNO_2_ system has been applied successfully in several dewaxing operations for short pipelines in the Campos Basin, Brazil [5]. The need to delay heat release for an extended period makes the adoption of this method challenging for lengthy pipes. Acetic acid, as the reaction activator, was emulsified together with NH_4_Cl. Almubarak et al. [6] proposed the emulsification of these two salts to treat deep water blockage.

Emulsification is employed to minimize environmental impacts and enhance energy efficiency as well [7,8]. In this context, the emulsification of heavy oil as an alternative fuel holds great promise in addressing environmental pollution and the energy crisis [7]. The critical factor lies in the micro-explosion phenomenon within the emulsion, which plays a vital role in improving fuel efficiency while simultaneously reducing harmful gas emissions. Gowrishankar and Krishnasamy [8] conducted a study indicating that a biodiesel-water emulsion exhibits greater potential for simultaneously reducing NOx and smoke emissions while enhancing engine performance.

Emulsification has been proven to be an effective method for controlling the reactivity of HCl as a strong acid [9,10]. Zhang et al. [10] reported that micro-emulsified HCl acid significantly reduced the reactivity of HCl; the emulsified acid dissolved only 1.23 g of the core after one hour, while the regular HCl dissolved 1.68 g in just 9 min. Emulsified acid has been introduced to retard the strong HCl acid and applied successfully to provide deeper penetration and overcome the drawbacks of regular HCl acid.

Recent research has been dedicated to developing microemulsions of acid in diesel, offering improved stability and better control over the acid’s reactivity [11,12,13]. These microemulsions are typically created using surfactants and cosurfactants in addition to the two immiscible fluids [14]. Wang et al. [12] successfully prepared an acid-in-aromatic solvent microemulsion by optimizing the surfactant-to-cosurfactant ratio. This microemulsion system exhibited smaller particle sizes and a slower mass transfer coefficient when compared to conventional emulsified acid at 60 °C. The mass transfer coefficient of the microemulsion acid was more than one order of magnitude smaller than that of the conventional emulsified acid.

Dantas et al. [13] developed a low-reactive microemulsion system consisting of Alkonat L90 (a non-ionic surfactant), n-butanol (a cosurfactant), kerosene (as the oil phase), and an HCl acid solution (as the aqueous phase) at different concentrations (1.5%, 5%, 10%, and 15% wt%). This microemulsion acid was investigated for enhanced oil recovery (EOR) and was found to significantly increase oil recovery, contributing to sustainability.

Microemulsions offer new possibilities for controlled acid treatments. Zhang et al. [10] developed a 70% acid-in-diesel microemulsion that included cetyltrimethylammonium chloride as a cationic surfactant, aliphatic ethoxylate alcohol AEO9 as a non-ionic surfactant, and butanol and n-octanol as cosurfactants. This microemulsified acid system exhibits small droplet sizes and excellent stability. Furthermore, when compared to a hydrochloric acid solution at the same concentration, it demonstrates remarkable corrosion inhibition properties. Additionally, this microemulsified acid system can tolerate high salinity levels, up to 80 g·L^−1^, making it a promising candidate for use as an acidizing fluid in various applications.

The pH value of a medium has a vital impact on the reaction rates for different reactions. Borate crosslinkers crosslink well with guar gum-based polymers at high pH conditions (≥10). At low pH conditions (in the acidic range), a reverse reaction occurs [15]. Enzymes need optimal pH environments to react for different purposes, including oilfield applications. Conventional enzymes exhibit limited activity in high pH systems (>8.5) such as those found in borates [16]. Studies have demonstrated that enzymes effectively degrade gels in low to neutral pH fluids [17]. The reaction of TCFs of interest in this study can be activated using acids; therefore, increasing the pH values of TCFs is anticipated to impact their reactivity.

The reaction of TCFs is associated with the generation of thermal and kinetic energies, which are of great interest in research and field applications. Recently, TCFs have been proposed for various upstream applications, including dewaxing, sandstone stimulation, water and condensate banking removal, fracture cleanup, EOR, foamed acids, and foamed fracturing fluids [18,19,20,21,22,23,24,25,26,27,28,29,30,31,32]. The upstream operations are often characterized by high downhole temperatures, one of the triggering reaction mechanisms of TCFs. The current study aims to assess the effectiveness of emulsification and pH adjustment as techniques to control TCF reactivity and their potential to contribute to TCF reaction sustainability. This study examined the effectiveness of these techniques in controlling the reactivity of TCFs, considering only temperatures as a triggering mechanism for TCF reactions.

## 2. Results Analysis

### 2.1. Reactivity of Emulsified TCFs in Diesel

To assess the reactivity of emulsified TCFs, autoclave testing was carried out where the pressure and heat (temperature increase) generated from the TCF reaction were monitored for emulsified TCFs in diesel systems compared to the neat TCFs. The effect of emulsifier type and concentration, reaction triggering temperature, and TCF fraction volume on the reactivity of TCFs were investigated.

### 2.2. Reactivity of Emulsified TCFs Versus Neat TCFs

**Figure 1:**
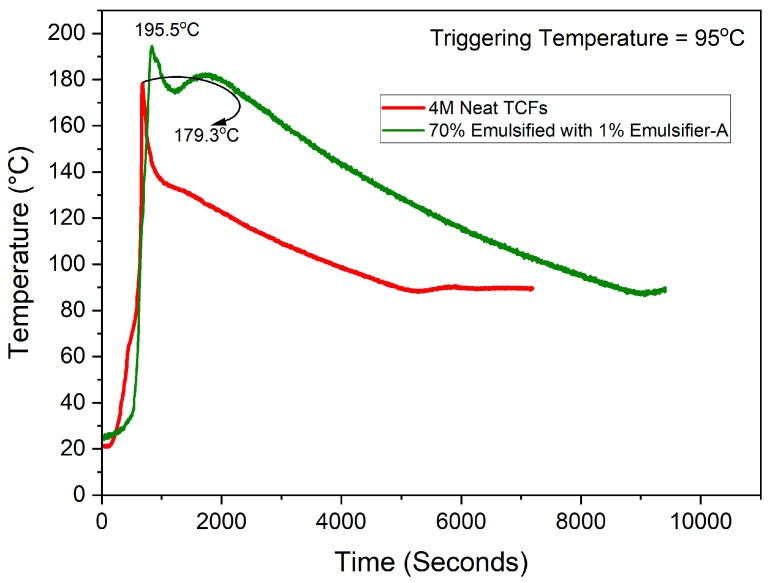
Effect of emulsification on the heat generated from the reaction of 70% individual emulsified 4M TCFs compared to neat 4M TCFs at 95 °C.

The reactivity of TCFs is assessed by the heat and gas generated, which is reflected in changes in temperature and pressure, respectively. Figure 1 illustrates the comparison of reactivity between individual emulsified 4M TCFs in diesel using Emulsifier-A and the neat 4M system at 95 °C. For the sake of simplicity and comparative purposes, we assumed ideal gas conditions and neglected the contribution of excess heat from the emulsification to the generated pressure. Figure 1 demonstrates that the heat generated by the emulsified system, as reflected in the temperature change, was higher (T_max_ = 195.5 °C) than that generated by the neat TCFs (T_max_ = 179.3 °C). The higher temperature increase in the emulsified TCFs is attributed to the lower specific heat capacity for diesel (2050 J/kg·C) compared to that for water (4184 J/kg·C). As known from the heat transfer equation, the change in temperature is inversely proportional to the specific heat capacity (ΔT ∝ 1/Cv). Moreover, the results showed that emulsification resulted in higher heat generation and retention compared to the neat system, clearly indicating that emulsification contributes to improved heat sustainability.

The reactivity of emulsified and non-emulsified TCF systems was assessed based on the pressure resulting from the generated nitrogen. Reactivity for a gas-producing reaction can be determined using the ideal gas law:

(2) $$\mathrm{PV}=\mathrm{nRT}\;\mathrm{and}\;C=\frac{n}{V}=\frac{1}{RT}P\;\Rightarrow \;\frac{dC}{dt}=\frac{1}{RT}\;\frac{dP}{dt}$$

*P* represents the pressure in Pascals (Pa), *V* is the volume in liters (L), *n* is the number of moles, *R* is the gas constant (J·mol^−1^·K^−1^), and *T* is the temperature in Kelvin (K). So, the value of $\frac{dP}{dt}$ can be determined from the generated pressure data and with this information, initial reaction rates.

**Figure 2:**
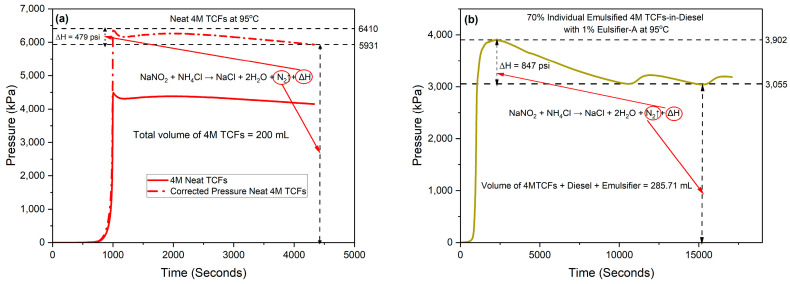
Reactivity of (**a**) 4M neat TCFs versus (**b**) 70% individual emulsified 4M TCFs at 95 °C with emulsifier-A.

In this study, we considered the maximum generated peaks as the ultimate reactivity of any system examined due to the gradual increase in temperature to the designated level. Figure 2 shows the pressure curves generated from neat 4M TCFs and 4M TCFs emulsified in diesel with 1% Emulsifier A at 95 °C. For the neat system, the pressure generated was corrected based on the P_1_V_1_ = P_2_V_2_ relation to match the volume of the emulsified system of 285.7 mL. The neat TCF system exhibits a lower peak-plateau difference, primarily due to the generated heat, compared to the emulsified TCFs systems. However, it resulted in higher pressure due to nitrogen generation when compared to the emulsified 4M TCFs with Emulsifier-A. The higher peak-plateau difference in the emulsified systems can be attributed to the previously mentioned higher specific heat capacity of diesel. The results demonstrate that achieving a complete reaction in the emulsified 4M TCFs, as is the case with Emulsifier-B addressed in the next section, is associated with higher pressure and heat generation compared to the neat TCFs system. This, in turn, enhances the efficiency of TCF reactions and overall sustainability.

### 2.3. Effect of Emulsifier Type

**Figure 3:**
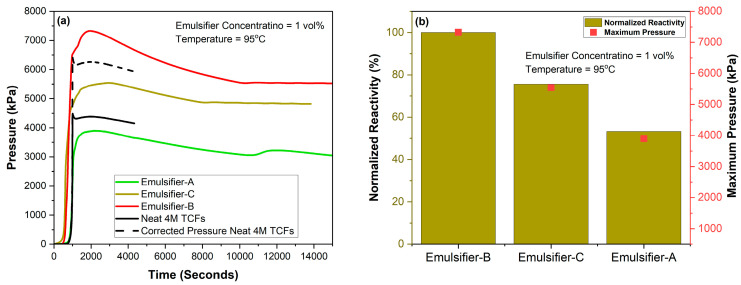
Reactivity of 4M neat TCFs and 70% individual emulsified 4M TCFs in diesel with different emulsifiers at (1%) and 95 °C (**a**) pressure and (**b**) normalized reactivity.

**Figure 4:**
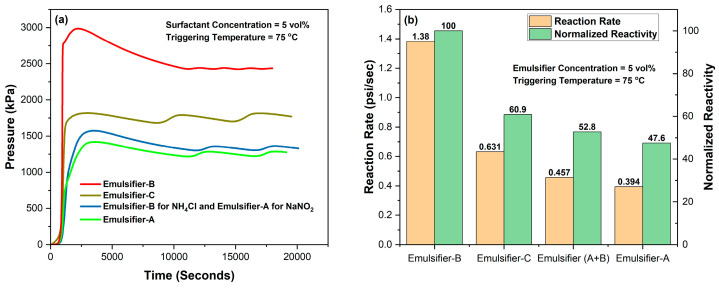
Reactivity of 70% individual emulsified 4M TCFs in diesel with different emulsifiers at (5%) and 75 °C, (**a**) pressure and (**b**) normalized reactivity and reaction rate.

**Figure 5:**
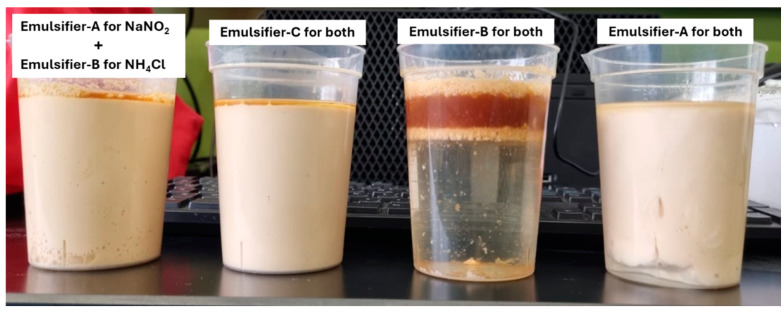
Photos of different emulsion systems with 5% of emulsifiers following autoclave testing at 75 °C.

The impact of emulsification on controlling the reactivity of individual emulsified 4M TCFs at a concentration of 70% in diesel, using the emulsifiers of interest in this study at both 1% and 5%, compared with neat 4M TCFs, was examined at temperatures of 95 and 75 °C, as shown in Figure 3 and Figure 4, respectively. The results revealed that the emulsion prepared using Emulsifier-B exhibited the highest reactivity (indicating the worst emulsification), while the emulsion prepared using Emulsifier-A exhibited the lowest reactivity (indicating the best emulsification). The same trend of performance was observed for emulsions prepared with 1 vol% and 5 vol% emulsifiers. These results are supported by the photos taken after the testing of emulsions with a 5 vol% emulsifier, which clearly showed a full separation of the emulsion prepared using Emulsifier-B (Figure 5). The lowest reactivity control observed for TCF emulsion with Emulsifier-B might be attributed to the positive charge nature of this emulsifier, while the best reactivity control obtained for emulsion with Emulsifier-A might be attributed to the high pH value of this emulsifier (1% Emulsifier-A in DI water, pH = 10). Based on viscosity measurements, which indicated the highest viscosity for the NaNO_2_ emulsion with Emulsifier-B and the highest viscosity for the NH_4_Cl emulsion with Emulsifier-A, we examined the NH_4_Cl/NaNO_2_ emulsions with emulsifiers (A and B), as shown in Figure 3. This system exhibited the second-highest control of reactivity. Based on the results obtained, Emulsifier-A was chosen for further investigation into various parameters that affect the reactivity of TCF emulsion, including temperature, emulsifier concentration, and TCF volume fraction.

### 2.4. Effect of Concentration, Temperature, Volume Fraction, and Agitation

The effect of concentration, temperature, volume fraction, and agitation on controlling the reactivity of the TCFs in diesel emulsions with Emulsifier-A will be discussed in the following sections.

#### 2.4.1. Effect of Emulsifier Concentration

**Figure 6:**
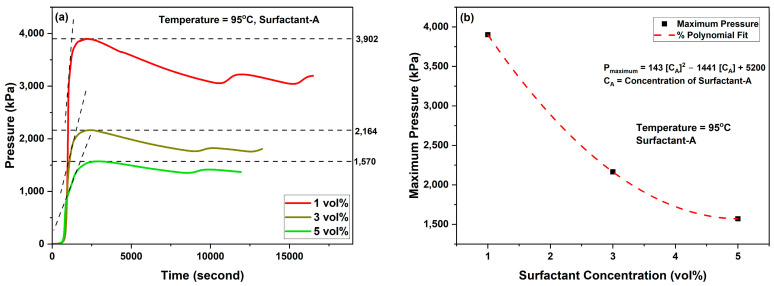
Generated pressure from emulsified 4M TCFs in diesel as a function of: (**a**) time and (**b**) Emulsifier-A concentration.

**Figure 7:**
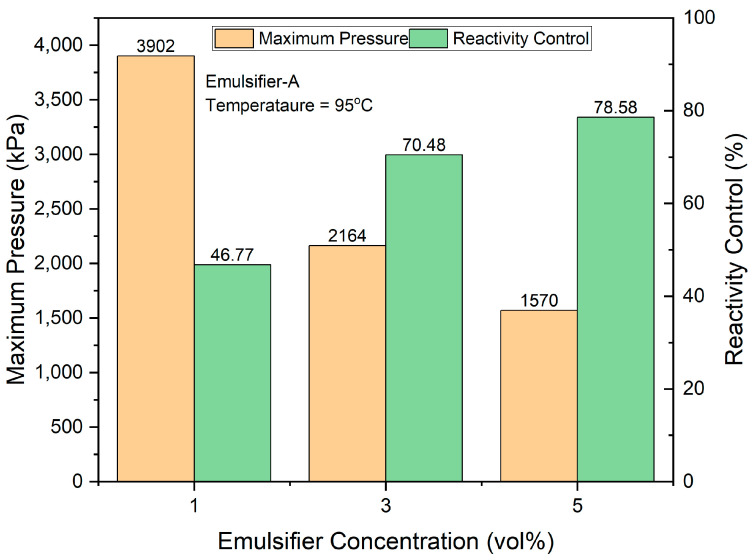
Effect of Emulsifier-A concentration on the reactivity of 4M TCFs emulsified in diesel at 95 °C.

The effect of emulsifier concentration on the reactivity of 70% of individual emulsified 4M TCFs with Emulsifier-A was investigated at a temperature of 95 °C, as shown in Figure 6. The results showed that as the emulsifier concentration was increased, the reactivity of TCFs decreased for the examined concentration of 1, 3, and 5 vol%. The reduction in the reactivity, defined as the maximum pressure obtained, is governed by a polynomial of second-order equation:

*P_max_* = 143 [*C_A_*]^2^ − 1441 [*C_A_*] + 5200 (3)

where *P_max_* is the maximum pressure in kPa, and *C_A_* is the concentration of Emulsifier-A in vol%. The control in the reactivity of TCFs, defined as the reduction in the reactivity compared to the reactivity of 4M TCF emulsion prepared with Emulsifier-B at 95 °C (*P_max_* = 7330 kPa), is governed by the following relation:

(4) $$\mathrm{Reactivity}\;\mathrm{Control}\;(\%)\;\;1-\frac{P_{max}(kPa)}{7330\;kPa}\times 100$$

where *P_max_* is the maximum pressure obtained at a certain concentration of Emulsifier-A. Figure 7 shows the reactivity control obtained at Emulsifier-A concentrations of 1, 3, and 5 vol%. When the emulsifier concentration was increased from 1 to 3 vol%, the reactivity control increased from nearly 47% to more than 70%. The highest reactivity control, exceeding 78%, was achieved at an emulsifier concentration of 5 vol%. The results indicate that the higher the emulsifier concentration, the greater the contribution to sustainability, as reactivity (reaction energy) is conserved for various downhole applications.

#### 2.4.2. Effect of Temperature

**Figure 8:**
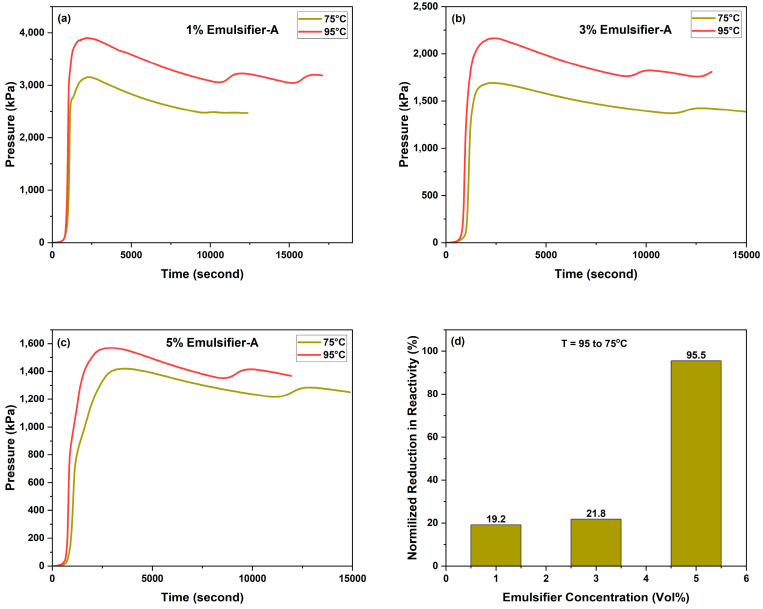
Effect of temperature on the reactivity of 70% Emulsified 4M TCFs in diesel with Emulsifier-A at (**a**) 1, (**b**) 3, and (**c**) 5 vol%, and (**d**) normalized reduction in reactivity for temperatures of 75 and 95 °C as a function of TCF concentration.

The influence of triggering temperature on the reactivity of 70% of individually emulsified 4M TCFs in diesel with Emulsifier-A at 1, 3, and 5 vol% was investigated, as shown in Figure 8. The reduction in TCF reactivity when decreasing the temperature from 95 to 75 °C was evident, especially for emulsifier concentrations of 1 and 3 vol% (19% and 21%, respectively). The reduction in TCF reactivity at an emulsifier concentration of 5 wt% was less than that obtained for emulsifier concentrations of 1 and 3 vol% by a factor of two.

**Figure 9:**
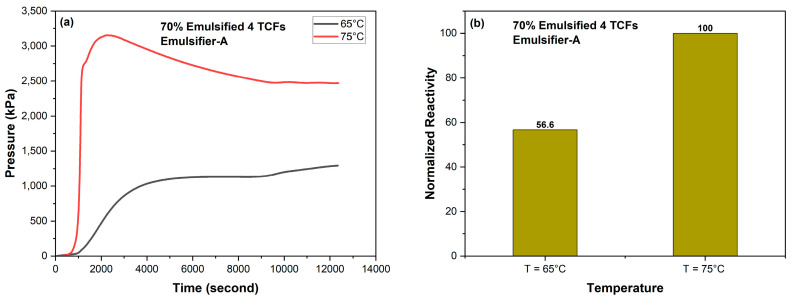
Effect of temperature on the reactivity of 70% 4M TCFs emulsified in diesel with Emulsifier-A at 1 vol%, (**a**) pressure and (**b**) normalized reactivity.

Figure 9 shows the effect of temperature on the reactivity of 70% 4M TCFs emulsified in diesel with 1 vol% Emulsifier-A at 65 and 75 °C. It was found that temperature had a significant impact on TCF reactivity, where decreasing the temperature by 10 degrees (from 75 to 65 °C) resulted in a reduction of TCF reactivity by more than 43%.

#### 2.4.3. Effect of TCF Volume Fraction

**Figure 10:**
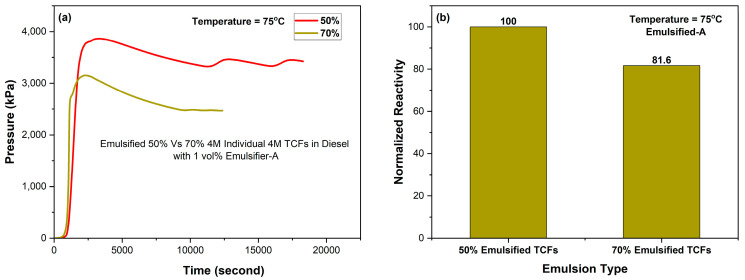
Effect of volume fraction on (**a**) pressure generated and (**b**) normalized reactivity of emulsified 4M TCFs in diesel with 1% Emulsifier-A at 75 °C.

Figure 10 shows the effect of TCF volume fraction on the reactivity of emulsified 4M TCFs in diesel using 1% Emulsifier-A at 75 °C. The results demonstrated that increasing the volume fraction of 4M TCFs from 50% to 70% resulted in a reduction of emulsified TCF reactivity by more than 18%. The results reveal that the higher the proportion of TCFs in the emulsion system, the better the reactivity control of TCFs, which is in favor of incorporating more TCFs and, thus contributing more to sustainability.

#### 2.4.4. Effect of Emulsification Method and Agitation

**Figure 11:**
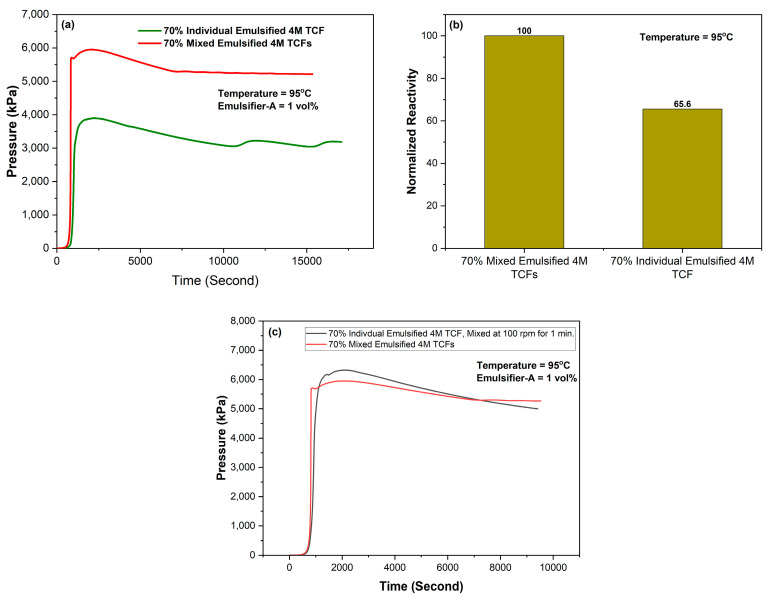
Effect of the emulsification method on the reactivity of 4M TCFs with 1 vol% Emulsifier-A at 95 °C (**a**) individual versus mixed emulsification, (**b**) normalized reactivity, and (**c**) individual emulsified followed with mixing versus mixed emulsified system.

The effect of the emulsification method on the reactivity of TCFs at 95 °C was examined using 70% individual and mixed 4M TCFs with Emulsifier-A, as shown in Figure 11. Individual emulsification provided better control over TCF reactivity, resulting in a reduction of nearly 35% compared to the mixed emulsion.

The impact of agitation on the reactivity of TCFs was examined by comparing 70% individually emulsified 4M TCFs in diesel with mixed emulsified 4M TCFs, as shown in Figure 11. The results revealed that agitation allowed the individual TCF-encapsulated droplets to come into contact, ultimately triggering the reaction. This is reflected in the significant pressure increase shown in Figure 11. These findings highlight the importance of avoiding agitation of emulsified TCFs, particularly during high-shear injection. To prevent interaction among TCF components during the injection stage, the use of a spacer between them would be more beneficial.

#### 2.4.5. Impact of pH and Initial Pressure on the Reactivity of Neat TCFs

**Figure 12:**
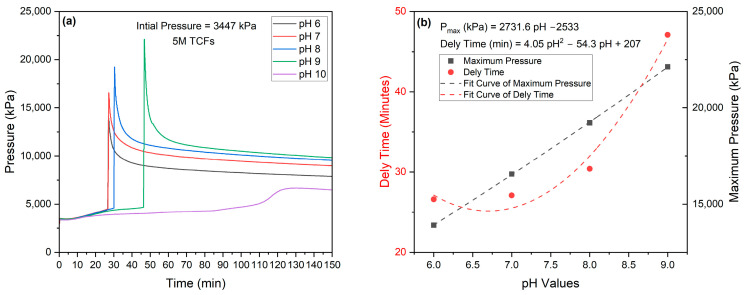
(**a**) Effect of pH on generated pressure by 5M TCFs and (**b**) relationship between TCF pH values and maximum pressure.

**Figure 13:**
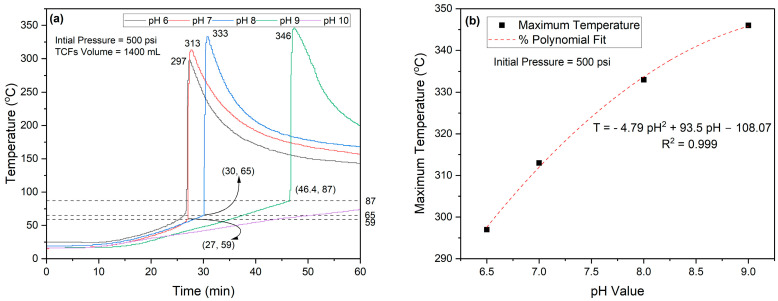
(**a**) Effect of pH on generated heat by 5M TCFs and (**b**) relationship between TCF pH values and maximum temperature.

Ammonium chloride is an acidic salt because it is derived from a weak base (ammonia, NH_3_) and a strong acid (hydrochloric acid, HCl) [19]. Sodium nitrite is a salt derived from a strong base (sodium hydroxide, NaOH) and a weak acid (nitrous acid, HNO_2_). The equimolar solution of NH_4_Cl and NaNO_2_ (2–4M) has pH values of 6.13–6.34 [4]. The reaction of thermochemical fluids (TCFs) of interest in this study is triggered either by acids or heat. Due to this inherent property of TCFs, their reactivity is highly influenced by pH. In this study, we investigated the effect of pH and initial pressure on the reactivity of TCFs using a 3 L autoclave reactor. A total volume of 1400 mL of an equimolar (5M) solution of NH_4_Cl and NaNO_2_ at a 1:1 volume ratio was used. The impact of pH values (6, 7, 8, 9, and 10) on the generated pressure and heat is shown in Figure 12 and Figure 13, respectively. The results showed that the pH values linearly influenced the generated pressure until pH = 9, as given by the following relation:

*P_peak_* (kPa) *=* 2731.6 pH − 2533 (5)

where *P_peak_* represents the maximum generated pressure at a certain pH value. The results also showed that increasing pH values tended to extend the delay time to start the TCF’s reaction, as given by the polynomial equation:

*Delay Time* (min) *=* 4.05 pH^2^ − 54.3 pH + 207 (6)

**Figure 14:**
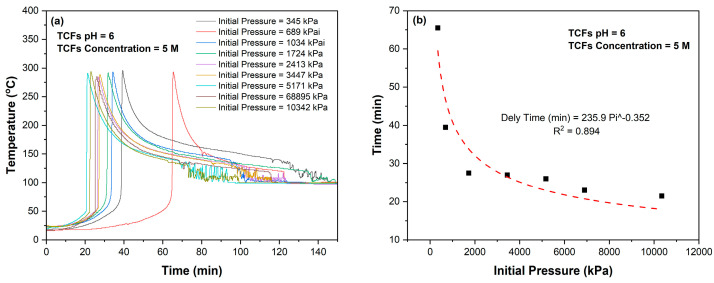
(**a**) Effect of initial pressure on delay time of 5M TCFs reaction and (**b**) relationship between initial pressure and time to trigger TCFs reaction.

Figure 14 shows the impact of initial pressure on the time required to trigger the reaction. The delay time is given by the following relation:

*Delay time* (min) = 235.9 *P_i_^*−0.352 (7)

where *P_i_* represents the initial pressure in kPa.

The results showed that both initial pH values of TCF and initial pressure have their impact on the reactivity of the TCF. These findings should be utilized in designing TCF systems for different applications.

## 3. Discussion

This study revealed that the reactivity of TCFs can be effectively controlled using emulsification, where Emulsifier-A exhibited the best performance. The higher the emulsifier concentration, the better the reactivity control of TCFs. Upon the complete reaction of emulsified TCFs in diesel, as with the case of Emulsifier-B, a higher generation of pressure and temperature was reported when compared to neat TCFs. Emulsification of TCFs also prevents the premature reaction of TCFs, which results in chemical and energy waste. These findings indicate that the emulsion of TCFs will contribute to sustainability. The data generated from the reactivity of emulsified TCF systems, such as reactivity control, should be utilized to optimize applications of TCFs in various downhole conditions. The type of emulsified TCF in terms of emulsifier concentration should be considered based on different well temperatures. Upon placement of the emulsified TCF system, a demulsifier, including acids, might be injected to break the emulsion and activate the reactivity of TCFs for a specific downhole application.

Another method to control the reactivity of TCFs and ultimately enhance the outcomes of TCFs in industry is by adjusting the pH of TCFs. Increasing the pH of TCFs was associated with a delayed reaction time and an increase in pressure and temperature in a sequence of TCF reactions. Additionally, the initial pressure was found to influence the triggering temperature and time required to start the TCF reaction. Table 1 gives the effect of pH and initial pressure on the triggering temperature, generated pressure, and time required to trigger the TCF reaction. The study findings presented in Table 1 can be utilized for different downhole applications based on well temperature and pressure to accurately predict the time required to start the TCF reaction at different downhole temperatures and pressures, enhancing the success of TCF applications.

Based on these findings, the strategy of managing the reactivity of TCFs through the elevation of pH proves to be effective at low depths. Nevertheless, at greater depths characterized by elevated initial pressures, the emulsification method appears as a more effective approach.

## 4. Materials and Methods

### 4.1. Materials

ACS reagent-grade sodium nitrite and ammonium chloride utilized in this study were obtained from Sigma-Aldrich (Steinheim, Germany) and Sigma-Aldrich Handels (Vienna, Austria), respectively. Deionized water (DI) with a resistivity of 18.2 MΩ·cm was used to prepare the salt solutions. An ACS-grade reagent sodium hydroxide obtained from Honeywell (Malmö, Sweden) was prepared at 6M and used to adjust the pH of NaNO_2_/NH_4_Cl solutions. The concentrated HCl acid (Muskegon, MI, USA) and NaOH (Honeywell, Malmö, Sweden) used to adjust the pH of TCFs were ACS reagent-grades from Fisher Scientific (Waltham, MA, USA). Three emulsifier systems, named Emulsifier-A, Emulsifier-B, and Emulsifier-C, with compositions detailed in Table 2, were obtained from local providers and utilized in this study to prepare TCFs in diesel emulsions. Each emulsifier contains both a surfactant and a cosurfactant. The relatively high pH value of 1 vol% of Emulsifier-A in DI water (9.51) is likely due to the presence of amine coco-alkyl acetate. In contrast, the low pH of Emulsifier-C is linked to the presence of acetic acid. Emulsifier-B shows a pH value of 4.74, which is attributed to the inclusion of a buffer system (acetic acid/acetates). A commercial diesel with a density of 0.78 g/cm^3^ was used to prepare the emulsions of interest.

### 4.2. Equipment and Methodology

**Figure 15:**
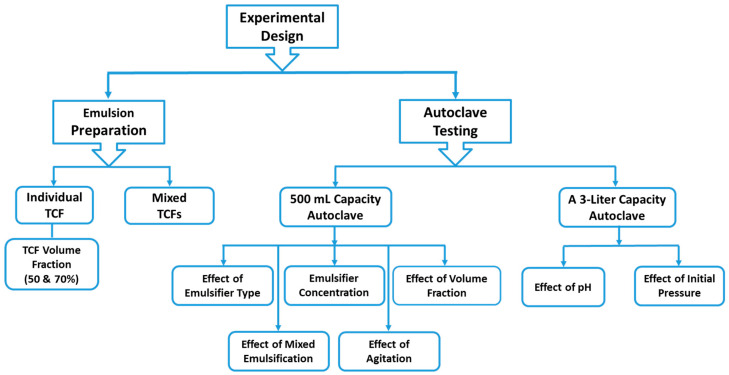
A schematic representation of the experimental work performed in this study.

The experimental work, as illustrated in Figure 15, involved emulsion preparation and autoclave testing. The autoclave testing was divided into the reactivity study of emulsified TCFs and the investigation of the impact of pH and initial pressure on the reactivity of non-emulsified TCFs.

### 4.3. Preparation of Emulsions

The preparation of TCFs in diesel emulsions was done using a high shear rate Silverson L5M-A model mixer (Silverson, Chesham, UK). TCFs in oil emulsions at 70/30 and 50/50 vol% were prepared by first dissolving the emulsifier at concentrations of 1, 3, and 5 vol% in diesel and allowing them enough time to mix thoroughly by agitating the mixture at around 3000 rpm for 2 min. The aqueous TCF phase was gradually added to the hydrocarbon phase of diesel, and the solutions were allowed to mix at a high speed of 10,000 rpm for 10 min to ensure the formation of emulsion. The emulsions were prepared at ambient temperature.

### 4.4. Emulsification Method and Agitation

Two emulsification methods were investigated in this study: one involving the individual emulsification of the TCF components (NaNO_2_/NH_4_Cl), and the other emulsifying them as a combined mixture. The 70% individually emulsified 4M TCFs in diesel were stirred at 100 rpm for 1 min before being subjected to autoclave testing.

### 4.5. pH Measurement

A Thermo Scientific™ Orion Star™ A111 Benchtop pH Meter (Jakarta, Indonesia) was used to measure pH values of the TCFs. The pH values of the investigated TCFs were adjusted using 6M NaOH (Honeywell, Sweden) solution.

### 4.6. Autoclave Testing

In this study, two different autoclaves were employed to study the reactivity of TCFs: one for investigating the impacts of emulsification and the other for examining the influence of pH. The OLT-HP-500 micro-mechanical mixing autoclave was used to examine the effect of emulsification on the reactivity of thermochemical fluids of interest in terms of generated temperature and pressure. The kettle body of the autoclave is Hastelloy, and it has a capacity of 500 mL and can tolerate 5000 psi (34,474 kPa) pressure and 500 °C temperature. It has an LCD where you can set and monitor the temperature. A pressure tracking device is connected to the system to monitor pressure generated as a function of time. All tests conducted in the autoclave were based on mixtures of a 1:1 ratio of NH_4_Cl to NaNO_2_.

The other autoclave reactor used in this study has a capacity of 3 L and can withstand a temperature of up to 500 °C and a pressure of 10,000 psi (68,947 kPa). The reactor is equipped with software controls for monitoring and tracking both pressure and temperature. This particular autoclave system was utilized to explore how the pH levels affect the reactivity of TCFs. A total volume of 1.4 L, consisting of equimolar (5M) NH_4_Cl and NaNO_2_, was used, and the pH values under examination for the TCFs were 6, 7, 8, 9, and 10. This study also delved into the impact of the initial pressure provided by nitrogen gas at various levels, including atmospheric pressure (14.7 psi, 101.4 kPa), 500, 750, 1000, and 1500 psi (10,342 kPa), on the reactivity of the NH_4_Cl/NaNO_2_ system when activated by heat. The heating rate is 4 °C/min. At the start of the reaction, a significant increase in temperature and pressure is observed on the tracking screen. The peaks of pressure and temperature are considered to be the maximum output of the reaction.

## 5. Conclusions

Extensive and detailed experimental work was carried out to assess the impacts of emulsification and pH adjustment on the reactivity of TCFs. A summary of the main findings is given below.

- Both emulsification and pH adjustment have the potential to promote sustainability by controlling the reactivity of TCF reactions.
- The reactivity of emulsified TCFs was found to be influenced by the emulsification method, emulsifier concentration, TCF volume fraction, and triggering temperature.
- The method of individual emulsification provided better reactivity control than the mixed emulsification method.
- Among the studied emulsifiers used as emulsifiers, Emulsifier-A demonstrated the most effective control of the reactivity of emulsified TCFs in diesel.
- The high pH values of TCFs (up to pH = 9) were found to positively influence the generated pressure, temperature, and time to trigger TCF reaction.
- Increasing initial pressure tended to decrease the time to trigger the reaction.
- The findings from the emulsification and pH adjustment study can be utilized to optimize various downhole applications of TCFs, enhancing the efficiency of TCF reactions and success rates.
- The strategy of managing the reactivity of TCFs through the elevation of TCF pH proves effective at low depths. Nevertheless, at greater depths characterized by elevated initial pressure, the emulsification method emerges as a more effective approach.

## Figures and Tables

**Table 1:** Effect of pH and initial pressure on the reaction triggering temperature, generated pressure, and temperature.

Exp # 1	Molarity	Initial pH	Initial Pressure (kPa)	Triggering Temperature (°C)	Max Pressure (kPa)	Time to Triggering Temperature (min)
1	5	9	101.4	115	11,928	73
2	5	9	3447	86	22,125	46
3	5	8	101.4	105	14,010	30
4	5	8	3447	65	19,223	30
5	5	6	3447	72	13,907	26
6	5	7	3447	59	16,561	27
7	5	8	3447	65	19,223	30
8	5	10	3447	170	6660	>120 min (Partial reaction)

**Table 2:** Composition, ionic properties, and pH values of the emulsifiers used in this study.

Emulsifier-A		Emulsifier-B		Emulsifier-C	
Component	Function	Component	Function	Component	Function
Tall oil fatty acid amine	Surfactant	Amines, tallow alkyl, acetates	Surfactant	Fatty Acid amine	Surfactant
Acetic aid		Heavy aromatic naphtha		Ethylene glycol	Cosurfactant
Ethylene Glycol	Cosurfactant	Ethane-1,2-diol	Cosurfactant	Heavy aromatic petroleum naphtha	To reduce an emulsion viscosity
Amine Coco-alkyl Acetate		Acetic acid		Acetic acid	
		1,2,4 trimethylbenzene		Naphthalene	
Ionic Properties of Emulsifiers					
Non-ionic		Positively charged		Negatively charged	
pH values of 1 vol% Emulsifier in D.I. Water					
9.51		4.74		1.62	

## Data Availability

All data generated or analysed during this study are included in this published article.
